# Deep Learning for Skin Melanocytic Tumors in Whole-Slide Images: A Systematic Review

**DOI:** 10.3390/cancers15010042

**Published:** 2022-12-21

**Authors:** Andrés Mosquera-Zamudio, Laëtitia Launet, Zahra Tabatabaei, Rafael Parra-Medina, Adrián Colomer, Javier Oliver Moll, Carlos Monteagudo, Emiel Janssen, Valery Naranjo

**Affiliations:** 1 Skin Cancer Research Group, INCLIVA, 46010 Valencia, Spain; 2 Faculty of Medicine, Universitat de València, 46010 Valencia, Spain; 3 Instituto Universitario de Investigación en Tecnología Centrada en el Ser Humano, HUMAN-Tech, Universitat Politècnica de València, 46022 Valencia, Spain; 4 Departmentof Artificial Intelligence, Tyris Tech S.L., 46021 Valencia, Spain; 5 Department of Pathology, Fundación Universitaria de Ciencias de la Salud, Hospital San José, 111411 Bogotá, Colombia; 6 Department of Pathology, Instituto Nacional de Cancerología, 110911 Bogotá, Colombia; 7 Department of Pathology, Stavanger University Hospital, 4068 Stavanger, Norway; 8 Department of Chemistry, Bioscience and Environmental Engineering, University of Stavanger, 4036 Stavanger, Norway

**Keywords:** skin, cancer, melanoma, melanocytic tumors, dermatopathology, computational pathology, deep learning, classification, segmentation, computer-aided diagnosis

## Abstract

**Simple Summary:**

Deep learning (DL) is expanding into the surgical pathology field and shows promising outcomes in diminishing subjective interpretations, especially in dermatopathology. We aim to show the efforts of implementing DL models for melanocytic tumors in whole slide images. Four electronic databases were systematically searched, and 28 studies were identified. Our analysis revealed four research trends: DL models vs. pathologists, diagnostic prediction, prognosis, and regions of interest. We also highlight relevant issues that must be considered to implement these models in real scenarios taking into account pathologists’ and engineers’ perspectives.

**Abstract:**

The rise of Artificial Intelligence (AI) has shown promising performance as a support tool in clinical pathology workflows. In addition to the well-known interobserver variability between dermatopathologists, melanomas present a significant challenge in their histological interpretation. This study aims to analyze all previously published studies on whole-slide images of melanocytic tumors that rely on deep learning techniques for automatic image analysis. Embase, Pubmed, Web of Science, and Virtual Health Library were used to search for relevant studies for the systematic review, in accordance with the Preferred Reporting Items for Systematic Reviews and Meta-Analyses (PRISMA) checklist. Articles from 2015 to July 2022 were included, with an emphasis placed on the used artificial intelligence methods. Twenty-eight studies that fulfilled the inclusion criteria were grouped into four groups based on their clinical objectives, including pathologists versus deep learning models (*n* = 10), diagnostic prediction (*n* = 7); prognosis (*n* = 5), and histological features (*n* = 6). These were then analyzed to draw conclusions on the general parameters and conditions of AI in pathology, as well as the necessary factors for better performance in real scenarios.

## 1. Introduction

Cutaneous tumors are the most common type of cancer. The bulk of deaths in this category are caused by melanoma, although it counts for only 1% of skin tumors. The histopathological diagnosis can sometimes be challenging, and it has demonstrated a significant discrepancy in pathologists’ interpretations [1,2,3]. The histopathological interpretation prevails as the gold standard for tumor diagnosis in a standard light microscope. Despite current genetic and epigenetic advances that have helped to understand the pathophysiology of these melanocytic tumors, some still remain undeciphered completely and represent a significant challenge in diagnosing and determining prognostic factors [4]. For instance, errors in cancer diagnosis might happen in as many as 11.8% of all cytologic–histologic specimen combinations [5]. The number of errors poses a serious issue for society as a whole, in addition to the patients.

In Digital Pathology (DP), the glass slide can be digitized to produce a high-resolution image resembling the one from the microscope, called Whole-Slide Image (WSI). According to studies, WSIs can be used for primary diagnosis just as successfully as a microscope [6].

A new chapter in pathology has been made possible by DP, including telediagnosis and the use of Artificial Intelligence (AI) to analyze histological images [5,7]. The implementation of AI strategies in health care has been a subject of great attention because it has the potential to augment the efficiency of experts. Regardless of whether the data chosen by the algorithm are deemed significant or not to the human eye, Deep Learning (DL) applications can leverage large amounts of data (including high-definition images) to identify important patterns and make accurate predictions [8]. All kinds of work in tumor pathology can be carried out using DL-based algorithms, including tumor diagnosis, subtyping, grading, staging, and prognosis prediction, as well as identifying pathological features and genetic changes. As AI can help improve diagnostic accuracy and objectivity, pathologists can spend more time on high-level decision making and integrating DL biomarkers into clinical workflows [9,10]. Additionally, the use of AI models in DP brings the promise of reducing pathologists’ workload, as well as diagnosis uncertainty for ambiguous lesions such as spitzoid melanocytic tumors.

To the best of our knowledge, this study is the first that aims to analyze the published research works of DL methods for automatic image analysis of melanocytic tumors’ WSIs exclusively. The chosen studies were reviewed and analyzed from both an engineering and a medical perspective by way of a Systematic Review (SR), in compliance with the Preferred Reporting Items for SRs and Meta-Analyses (PRISMA) standards [11]. In this work, we propose to evaluate the significance of clinical data in the diagnosis and/or prognosis and the performance of the established pipeline using DL. These are then used to discuss and draw conclusions according to their specific clinical aims and highlight the related issues of these methods to become more effective in real scenarios.

## 2. Materials and Methods

### 2.1. Literature Search Strategy

Embase, Pubmed, Web Of Science (WOS), and Virtual Health Library (VHL) were used to search for histopathological image analysis of melanocytic tumors, with a focus on the applied AI techniques and including papers from 2015 up to July 2022. The PRISMA [11] guidelines were followed during data extraction, analysis, and reporting. The words used for the search were the following: "(Nevi and Melanomas) AND (neural networks, computer OR DL) AND (pathology)" according to Emtree terms for Embase, MesH terms for PubMed, Keywords for WoS, and Decs/MesH terms for VHL.

### 2.2. Study Eligibility and Selection

For the SR, the inclusion criteria were studies on WSIs of melanocytic tumors using DL models for image analysis and processing. As for the exclusion criteria, articles were removed from this review if:

i. No DL-based methods were used;
ii. The writing language was different than English;
iii. The analyzed tissues containing melanoma were other than skin (e.g., lymph node metastasis and uveal melanoma);
iv. The used data sets were not of human origin.

Regarding the study selection, two reviewers, including one certified pathologist (AMZ) and one engineer with image analysis experience (LL), independently screened all the titles and abstracts from the publications and performed an eligibility assessment. For the selected articles, the data retrieved from each study were the following: general information regarding paper publication (author, publication year, and country), the aim of the proposed work, clinical and histopathological data, data preparation information (WSI scanners, magnification level, patch size, number of images, and image preprocessing steps), Ground Truth (GT), evaluation metrics (accuracy, sensitivity, specificity, and segmentation metrics), used GPU, and DL methodology (architectures, patch aggregation, etc.).

The quality of the publications was evaluated according to the Checklist for Artificial Intelligence in Medical Imaging (CLAIM) [12]. It is important to mention that one of the studies was a letter to the editor [13]. After reviewing its eligibility and discussing it with three reviewers, we decided that this publication would be included because it fulfilled the eligibility criteria despite being a research letter.

### 2.3. Study Analysis and Performance Metrics

The three most important categorization performance indicators selected for this study were accuracy, sensitivity, and specificity, when they were available. The amount of accurate and inaccurate predictions made by a classification model affects how well it performs. One of the most frequently used classification evaluation measures, accuracy, is calculated by dividing the total number of predictions given a data set by the number of right predictions. When the target classes in the data set are evenly distributed, accuracy is useful; nonetheless, it should not be utilized as a unique measurement. In contrast to accuracy, sensitivity and specificity are unaffected by imbalanced data. The algorithm diagnoses the proportion of cancer patients and computes the sensitivity with a better performance expression for a specific class. Opposite to sensitivity, specificity reports the portion of patients that did not have cancer but were predicted by the model as noncancerous.

## 3. Results

**Figure 1:**
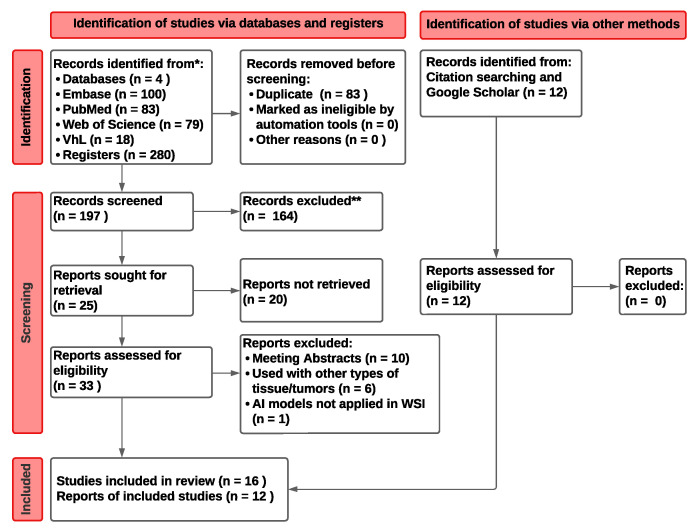
PRISMA flow diagram describing the search and selection process carried through for this systematic review [11].

As a result, 292 articles in total, including 100 from Embase, 83 from PubMed, 79 from WoS, 18 from VHL, and 12 from other sources (citation searching and Google Scholar), were found. Out of all these papers, 83 were duplicate studies that were eliminated. To establish if the remaining 209 studies matched the eligibility requirements, their titles and abstracts were carefully examined. The PRISMA flow diagram summarizing the included searches and databases is shown in Figure 1.

All in all, 28 studies were included according to the inclusion criteria and were categorized, after reviewing their objectives and methodology, into the four following groups, as shown in Table 1:

- DL models vs pathologists (*n* = 10), where the algorithm is compared with a group of pathologists apart from those who were in charge of GT;
- Diagnostic prediction (*n* = 7), where the algorithm demonstrates its performance differentiating different groups of melanocytic lesions (e.g., melanoma and nevus);
- Prognosis (*n* = 5), where the algorithm recognizes important characteristics to determine the patient prognosis, i.e., lymph node metastasis and disease-specific survival (DSS), among others;
- Histological features and Regions Of Interest (ROIs) (*n* = 6), where the algorithm identifies key histopathological ROIs for further diagnosis (e.g., mitosis, tumor region, and epidermis).

First and foremost, the number of data sets used as source data differed quite a bit. Indeed, 13 studies (46%) used only one internal institutional source [14,15,16,17,18,19,20,21,22,23,24,25,26], while five works (18%) [27,28,29,30,31] used open access repositories, such as The Cancer Genome Atlas (TCGA) [32] and the National Cancer Institute (NCI) Genomic Data Commons (GDC) [33], except for the work conducted by Zormpas et al. [31], who only used TCGA as their unique source. It is noteworthy that Phillips et al. [27] used the largest number of sources (*n* = 10 including TCGA). One of the studies (4%) did not describe the source that was used for the DL model [34], and the remaining 32% used at least two different data sources (see Table 1).

**Figure 2:**
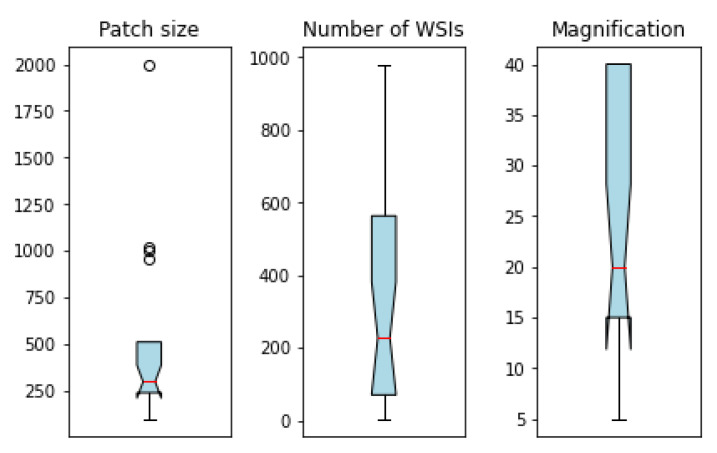
Minimum, median, and maximum amount of patch size, number of WSIs, and magnification. The median amount of each parameter is represented by a red vertical bar.

These publications differ greatly in terms of the number of WSIs employed, size of patches, and their magnification. For instance, the number of WSIs varies from 4 [22] to 981 [35] among studies. Figure 2 illustrates these variations by giving an overview of the minimum, median, and maximum amount of the used patch size, number of WSIs, and magnification in the reviewed articles.

All the studies used the pathologists’ interpretation as the GT. Out of these, 12 (42.9%) added heat maps to explain the approach of the algorithm [17,18,19,23,27,29,34,35,36,37,38,39].

Nine studies (31%) used clinical metadata as a part of the pipeline for the prediction of the algorithm [13,16,28,29,30,31,38,40,41]. All the studies in the prognosis group took clinical information into account, and two of them used a follow-up of 24 months [40,41]. In the ROI’s group, clinical information was not used for their predictions.

Below, we summarize these studies according to their objective, methodology, and results.

**Table 1:** Studiesincluded in the systematic review, divided into the four previously defined categories, along with their main parameters. Mag.: magnification level. n/a: not available. #: Number. xAI: studies that provide elements for explainable AI, e.g., GradCAMs or attention mechanism.

	Study	Year	Studied Structures	Mag.	# WSIs	Patch Size	Pre-Processing	DL Method	GPU Used	# Sources	Metadata	xAI
Comparison vs. pathologists	Ba et al. [42]	2021	Tumor	40×	781	256 × 256	Image quality review	CNN and random forest	n/a	2	no	yes
	Bao et al. [36]	2022	Tumor	40×	981	224 × 224	Random patch selection, structure-preserving color normalization	ResNet-152	NVIDIA GTX 2080Ti	3	no	no
	Brinker et al. [43]	2022	Tumor	n/a	100	n/a	n/a	ResNeXt50	n/a	n/a	no	yes
	Hekler et al. [14,15]	2019	Tumor	10×	695	n/a	n/a	ResNet50	n/a	1	no	no
	Phillips et al. [27]	2019	Tumor, dermis, and epidermis	40×	50	512 × 512	Subtraction	Modified FCN	NVIDIA GTX 1080 Ti	10 †	no	yes
	Sturm et al. [16]	2022	Mitosis	20×	102	n/a	n/a	n/a	n/a	1	yes	no
	Wang et al. [37]	2020	Tumor	20×	155	256 × 256	Random cropping to 224 × 224, data enhancement, and augmentation	VGG16	n/a	2	no	yes
	Xie et al. [28]	2021	Tumor, dermis, and epidermis	20×	701	224 × 224	Discard blank patches (Otsu)	ResNet50	n/a	3 †	yes	no
	Xie et al. [17]	2021	Tumor	n/a	841	256 × 256	Discard blank patches (Otsu)	ResNet50	NVIDIA TITAN RTX	1	no	yes
Diagnosis	Del Amor et al. [19]	2021	Tumor	10×	51	512 × 512	Discard blank patches (Otsu)	VGG16 with attention	NVIDIA DGX A100	1	no	yes
	Del Amor et al. [18]	2022	Tumor	5×, 10×, 20×	43	512 × 512	Discard blank patches and with less than 20% of tissue (Otsu)	ResNet18 with late fusion of multiresolution feature maps	NVIDIA GP102 TITAN Xp	1	no	yes
	Hart el al. [34]	2019	Tumor	40×	300	299 × 299	n/a	InceptionV3	4 NVIDIA GeForce GTX 1080	n/a	no	yes
	Höhn et al. [38]	2021	Tumor	n/a	431	512 × 512	Remove patches with more than 50% of background, random selection of 100 tiles per slide	ResNeXt50 with fusion model to combine patient data and image features	NVIDIA GeForce GTX 745	2	yes	yes
	Li et al. [29]	2021	Tumor, dermis, and epidermis	20×	701	224 × 224	Discard blank patches (Otsu)	ResNet50	n/a	2 †	yes	yes
	Van Zon et al. [20]	2020	Tumor	40×	563	256 × 256	Data augmentation	U-Net	NVIDIA 2080	1	no	no
	Xie et al. [21]	2021	Tumor	40×	312	500 × 500	Filter out background tiles	Transfer learning vs fully trained: InceptionV3, ResNet50, MobileNet	n/a	1	no	no
Prognosis	Brinker et al. [13]	2021	Tumor	n/a	415	256 × 256	n/a	ResNeXt50	n/a	3	yes	no
	Kim et al. [30]	2022	Tumor, inflammatory cells, and other	20×	305	299 × 299	n/a	Inception v3 with fivefold cross-validation	n/a	2 †	yes	no
	Kulkarni et al. [40]	2020	Tumor, inflammatory cells, and other	40×	n/a	500 × 500	Downsample to 100 × 100, nuclear segmentation with watershed cell detection	n/a	n/a	2	yes	no
	Moore et al. [41]	2021	Tumor, inflammatory cells, and other	40×, 20×	n/a	100 × 100	n/a	QuIP TIL CNN [44]	NVIDIA GP102GL [Quadro P6000]	2	yes	no
	Zormpas-Petridis et al. [31]	2019	Tumor, inflammatory cells, and other	20×, 5×, 1.25×	105	2000 × 2000 (20× WSIs)	n/a	Spatially constrained CNN with spatial regression, neighboring ensemble with softmax	NVIDIA Tesla P100-PCIE-16GB	1 †	yes	no
ROI/histological features	Alheejawi et al. [22]	2021	Tumor, inflammatory cells, and epidermis	40×	4	960 × 960	Divide patches into 64 × 64 blocks	ResNet50	NVIDIA GeForce GTX 745	1	no	no
	De Logu et al. [39]	2020	Tumor and healthy tissues	20×	100	299 × 299	Data augmentation, discard patches with more than 50% background	Inception-ResNet-v2	n/a	3	no	yes
	Kucharski et al. [23]	2020	Tumor	10×	70	128 × 128	Data augmentation, overlapping only for minority class to balance data set	Autoencoders	n/a	1	no	yes
	Liu et al. [24]	2021	Tumor	10×	227 ROIs ‡	1000 × 1000	Downscale magnification to 5×	Mask R-CNN	4 NVIDIA GeForce GTX 1080	1	no	no
	Nofallah et al. [25]	2021	Mitosis	40×	22	101 × 101	Data augmentation	ESPNet, DenseNet, ResNet, and ShuffleNet	NVIDIA GeForce GTX 1080	1	no	no
	Zhang et al. [26]	2021	Tumor	n/a	30	1024 × 1024	Data augmentation, color analysis for tissue-contained patch selection, normalization of patches to a uniform size, resize patches to 512 × 512	CNN, feature fusion	NVIDIA RTX 2080-12G	1	no	no

† At least one of the source institutions is open source, i.e., TCGA or NCI. ‡ Images are ROIs extracted from initial WSIs.

### 3.1. Deep Learning Models vs. Pathologists

This group has the highest number of studies (*n* = 10) in our research. All showed equal or better performance compared with the groups of pathologists who participated in the studies.

Most of the works in this category used a ResNet architecture or one of its variants as a basis to train their models. Brinker et al. [43] built an ensemble of pretrained ResNeXt50 CNNs to compare their classifier with 18 international expert pathologists in a task that discriminates melanomas from nevi. To do so, the authors leveraged both annotated and unannotated WSIs (i.e., both with and without the ROI delineation) and performed the predictions at patch level. The patches’ malignant scores were then averaged to obtain the WSI-level prediction. With a discordance of 13%, the algorithm had a better accuracy compared with pathologists in the slides without annotation (92% [SD = 1%] vs.90% [SD = 4%], respectively).

Moreover, Hekler et al. [14] performed the first head-to-head comparison of the classification results for randomly cropped images with 11 practicing histopathologists. The main goal of this study was to illustrate the benefit of DL techniques in medical diagnosis, especially with limited information between nevi and melanoma. The proposed CNN exceeded its performance with statistical significance (McNemar tests, *p* = 0.016) in respect to the pathologists with an accuracy of 68% (SD = 8%) vs 59% (SD = 5%), respectively. A previous study by the same author, using the same data set, showed that the algorithm had a discordance with histopathologists of 19%, similar to results seen in the literature between pathologists [15].

ResNet50 was employed in two articles by Xie et al. [17,28] as well. The first suggested utilizing a trust counting method to automatically diagnose melanoma, junctional nevi, intradermal nevi, and compound nevi. The patch preparation, patch-level model inference, and trusted computing approach for WSI diagnosis composed the three components of the diagnostic system. To obtain the classification probability at the slide level, the authors applied a trust counting method by averaging the weights of patches predicted as a given class with respect to all patches. The system’s effectiveness was then demonstrated by comparing results with 20 pathologists and attained an Area Under the Receiver Operating Characteristic (AUROC) of 98.6%, a sensitivity of 93.8%, and a specificity of 95.7% [28]. Additionally, Xie et al. proposed an interpretable diagnosis process in another work [17], performing WSI-level classification with a similar counting method from the patches’ inference results. To provide interpretable results, the authors generated heat maps by means of the Grad-CAM method to highlight the key feature regions of pathological images at the patch-level inference step. In this study, five responsible board-certified pathologists designated the lesion area and confirmed the labels that were obtained on the WSIs. The achieved accuracy at the best point was 93%, although for pathologists’ average point it was 73% [17].

It is important to highlight that most of the studies in this group performed a binary classification of the lesions under study (i.e., benign/malignant), and only three works also considered the performance for atypical melanocytic lesions [16,36,42]. In their work, Bao et al. [36] developed a DL-based fully automated diagnostic method to classify into these three classes, divided into patch prediction and patient diagnosis stages. While the first step was based on a ResNet-152 architecture to perform a patch-level classification, the second part consisted of aggregating the previous results for the final patient diagnosis. Specifically, the authors leveraged previous studies [45,46] to perform a patch voting aggregation strategy, where a WSI is assigned the class of the majority of patches. The results of the proposed framework were then compared with one junior pathologist and two senior pathologists. In diagnosing benign, atypical, and malignant melanocytic lesions, the technique outperformed pathologists in both the internal and external testing sets, reaching better F1-scores. The three pathologists reevaluated the type of melanocytic lesion in these patients after receiving the diagnosis results from the suggested method, which showed an improvement, particularly for the junior pathologist with statistically significant accuracy. This was carried out to confirm the clinical utility of the suggested method. Another study that included atypical lesions was that of Sturm et al. [16], who applied a DL algorithm previously developed for breast carcinoma mitosis cells based on a marker Phosphohistone-H3 (PHH3) [47], to melanocytic lesions. In this work, the authors aimed to demonstrate whether the initial mitosis algorithm could be used for cutaneous melanocytic tumors’ analysis on H&E-stained images. The original algorithm relied on an ensemble of networks trained on different data sets. The performance was compared with eight pathologists (two academic and six dermatopathologists) in two rounds: first without, and second with the help of a mitosis detection algorithm, with a washout period of at least two months in between. The overall concordance of the pathologists with the consensus diagnosis for all cases excluding nevoid melanoma (*n* = 89) appeared to be comparable with and without AI (89% vs. 90%). However, the concordance increased using AI in nevoid melanoma cases (*n* = 10) (75% vs. 68%).

In the pipeline of some studies, we found that two papers combined random forests with a CNN model [37,42]. Among them, Ba et al. [42] introduced a DL algorithm for discriminating melanoma from nevus. The proposed framework was conducted by integrating three modules: an ROI tissue extraction stage, a CNN-based melanoma patch detection step, and a slide-level classification module that considered atypical melanocytic tumors as well. In this approach, the random forest enabled the slide-level classification, taking as input the heat map of the patch-level prediction. Then, the algorithm was compared with seven dermatopathologists who used MPATH-DX [48] for their diagnosis. There was no statistical difference between the algorithm’s sensitivity and specificity and the main performance of the pathologists. A weighted error was used to reflect the fact that a false-negative result (failing to diagnose) was more detrimental than a false-positive result (making a melanoma diagnosis when it was not). The DL algorithm outperformed all except one dermatopathologist based on the weighted error scale, where the lower score presented a better diagnostic performance yielding a score of 1%, and the weighted error of the seven dermatopathologists ranged from 1% to 7%. In addition, Wang et al. [37] established a model for the detection of neoplasia in melanocytic tumors of the eyelid. While patch-level classification was based on a VGG16-based model architecture, WSI-level prediction consisted of a random forest ML algorithm on the features extracted from the visualization heat map. The outcome reached an accuracy of 98%, compared with the mean accuracy of seven pathologists of 92% ± 6.2%.

Finally, Phillips et al. [27] proposed to segment the epidermis, dermis, tumor areas, and essential structures for the Breslow thickness prognostic assessment. More precisely, the authors leveraged Long et al.’s FCN-style network implementation [49] and modified it to generate three levels of output granularity maps that they combined by means of a weighted and element-wise summation. To assess the accuracy of the model’s predictions, four pathologists measured the Breslow thickness with an inter-rater agreement of 87% with a fixed marginal kappa of 0.5.

### 3.2. Diagnostic Prediction

This group’s principal objective was to differentiate two types of melanocytic lesions. We found six of them, mainly focused on melanomas and nevus [18,19,20,21,29,38]. Hohn et al. [38] combined histologic features with patient data (age, sex, and anatomical site of the lesion) by means of fusion models in order to increase the accuracy in a binary classification task (melanoma/nevus). For the former, a baseline ResNetX50-based image classifier was trained, while the patient data classifier consisted of a random forest ensemble learning method. The reported results in this article confirm that patient data integration to CNN did not improve the accuracy.

In a study performed by Li et al. [29], the authors suggested an approach for melanoma detection and localization using WSIs of several melanocytic tumor types (melanoma, intradermal nevi, compound nevi, and junctional nevi). A ResNet50-based CNN model was first trained for patch-level inference, followed by the WSI-level prediction, where averaging the patches’ predicted scores showed a slightly better performance than counting the percentage of patches of each class. The system had an AUROC of 97% when using an average technique to assess the performance of melanoma categorization.

In parallel, Van Zon et al. [20] applied a U-Net architecture for patch-level semantic segmentation of tissues into melanoma, nevus, or negative for both. The authors then combined the resulting masks with their corresponding tissue patches to feed a CNN model to perform slide-level classification. The results on the melanoma data set have a sensitivity of 97% and 96%, and a specificity of 98% and 99%, respectively.

Additionally, Xie et al. [21] examined the precision of using Inceptionv3, ResNet50, and MobileNet on WSIs in separate and nonoverlapping patches. In total, 195 nevi and 117 melanoma were classified using two alternative training methods: transfer learning and fully trained models. In this study, tissue areas were manually delineated by three dermatologists and two pathologists for the purpose of analyzing the outcomes. The results demonstrate that three convolutional network models are capable of accurately classifying diseased skin images, with an accuracy ranging from 96.69% to 99.88% and an AUC of 99.44% to 99.99%. As can be observed, different models perform similarly while using the same training technique. The experiment demonstrated that in the classification of skin pathological image, the fully trained technique outperformed transfer learning.

Only three of the studies in developing predictions for diagnostic purposes included Spitz tumors. Del Amor et al. [19] proposed an attention-based weakly supervised method for spitzoid melanocytic lesion diagnosis in 51 WSIs. Specifically, the authors trained a VGG16-based feature extractor with an attention module [50] for aggregation, refined with Squeeze-and-Excitation blocks [51] for contextual information. The test results achieve an accuracy of 92.31% at a 10× magnification. Subsequently, a year later, Del Amor et al. presented a multiresolution framework to assess morphological features at different resolution levels and combine them to provide a more accurate diagnosis for Spitz tumors [18]. After optimizing the weights at a single resolution to enable transductive learning, the multiresolution model allowed to combine the perspective of three different resolutions (5×, 10×, and 20× magnification levels) by means of a late fusion concatenating the obtained feature maps. The experiments demonstrated that the proposed method outperformed single-resolution frameworks in Spitz tumor classification. The comparison of the results obtained by the model at 5× resolution and the proposed multiresolution framework showed that the latter outperformed with an AUC of 83% compared with 54%.

Finally, Hart et al. [34] applied a CNN based on the Inception V3 network architecture to distinguish between conventional and Spitz nevi. The model was trained at the patch level, both with WSIs curated by two certified dermatopathologists and noncurated ones. Then, the number of patches predicted as Spitz or conventional was tallied, and the overall slide prediction was computed with whichever category was more abundant. The classification accuracy of the 200 testing WSIs was 92%. Sensitivity was 85%, with a specificity of 99%. On a per-class basis, 99 of 100 conventional nevi were classified correctly (99%), compared with only 85% for Spitz nevi. Of the 16 misclassified WSIs, 94% were due to Spitz-type lesions being classified as conventional. When further exploring the false-positive calls, a strong edge effect was observed around the decision boundary, meaning that the incorrect calls were primarily driven by minor differences in the expected versus observed classes.

### 3.3. Prognosis

This group of studies focused on the prediction of prognosis (survival, metastasis, and genetic mutations) using WSIs as a source. All the studies used clinical metadata, and two registered a follow-up of 2 years ([40,41]).

Kulkarni et al. [40] proposed a DL method to predict visceral recurrence and DSS in patients with primary melanoma. They designed a deep neural network (DNN) architecture consisting of a CNN and a recurrent neural network (RNN). More precisely, the CNN enabled the extraction of high-dimensional features from the patches, and these features were then processed to identify spatial patterns. The concatenation of features with the RNN output to feed a fully connected layer then allowed to generate the final recurrence prediction. An ROC analysis showed that the predictor strongly correlates with Distant Metastatic Recurrence (AUC = 90.5%, 90%, and 88% in the two participating institutions), independent of tumor location or tumor type. The multivariable regression shows that the DNN predictor correlated with DSS when other clinical predictors were included as covariables with HR= 58.7 (*p* < 0.001).

Subsequently, Brinker et al. [13] developed a digital biomarker to predict lymph node metastasis from digitized H&E slides of primary melanoma tumors, based on Kulkarni et al.’s [40] cell feature extraction process. These were computed and combined with patient clinical information and fed into a multilayer perceptron classifier with a Squeeze-and-Excitation module [51], while a ResNeXt50-based CNN was also trained for patch-level feature extraction. Finally, all the information extracted in parallel was combined through a final classifier that gave a patch-level score, then averaged to give the final WSI prediction. A matched vs. unmatched analysis between Sentinel Node (SN)-positive and SN-negative cases was used for patient age, ulceration, and tumor thickness. The best accuracy was achieved in trained and tested unmatched cases, (61% ± 0.2) AUROC only using image features, with a sensitivity of 48% ± 14.2 and a specificity of 64% ± 11.1, respectively. The ANNs trained and tested on matched cases achieved (55% ± 3.5%) AUROC or less. The combination with the clinical features did not perform better, with an AUROC of 61% ± 0.4.

Regarding the importance of the tumor microenvironment as a prognostic feature, Zormpas et al. [31] described the importance of tumor infiltrating lymphocytes in DSS. The authors proposed a multiresolution hierarchical framework aiming to leverage both global and local context to improve cell classification. To do so, they first trained a spatially constrained CNN [52] (SC-CNN) at a higher resolution to detect and classify cells (cancer cells, stroma cells, lymphocytes, and epidermis cells). Then, they combined the cellular neighborhood information with that of regional tumor classification (cancer area, normal stroma, normal epidermis, lymphocyte cluster, and lumen/white space) on lower resolution images by means of a conditional random field [53] that connected single-cell nodes to regional classification results. The proposed model demonstrated the importance of spatial neighboring and global context, with an accuracy of 96.5% compared with that of SC-CNN alone (84.6%) on single-cell classification results. It also showed that a high ratio of lymphocytes to all lymphocytes within the stromal compartment (*p* = 0.026) and a high ratio of stromal cells to all cells (*p* < 0.0001 compared with *p* = 0.039 for SC-CNN only) are associated with poor survival in patients with melanoma.

Two years later, Moore et al. [41] used a previously validated software called Quantitative Imaging in Pathology to detect tumor-infiltrating lymphocytes (TIL), or QuIP TIL CNN, in early-stage melanomas. A Multivariable Cox proportional hazards analysis was performed using automated digital TIL analysis (ADTA), depth, and ulceration as covariables. It showed that the model contributed significantly to DSS prediction with a Hazard Ratio (HR) of 4.18 (Confidence Interval CI 1.51–11.58, *p* = 0.006) compared with the conventional TIL’s grading, depth, and ulceration made by pathologists; only depth contributed to the prediction. (HR = 1.40, CI 1.03–1.89, and *p* = 0.031). Within the validation set, depth, ulceration, T stage, and TIL grade correlated with DSS by univariable analysis; ADTA significantly exceeded with HR = 4.79, CI 1.74–13.22, and *p* = 0.002.

Targeting the prediction of genetic mutations using H&E WSIs, Kim et al. [30] used two approaches to predict BRAF mutations in melanoma. The first one used the Inception V3 architecture to predict the presence of BRAF mutation from WSI and the possible associated image features, and the second approach was detecting and quantifying nuclear differences from WSIs. More precisely, the former started by identifying ROI patches with an Inception-V3-based model before training the model for BRAF mutation prediction on ROI patches only, with transfer learning. The final slide prediction was then computed by averaging the patches’ probabilities. In parallel, the latter approach consisted of annotating nuclei on patches and then measuring nuclear features and relied on two previously developed tools [54,55]. Here, Kim et al. combined these methods with clinical information and showed that they outperformed the predictive power of any single model, achieving an AUC of 89% (95% CI = 0.75–1) and an AUC of 79% (95% CI = 0.55–1) on the internal and TCGA data sets, respectively.

### 3.4. Histological Features and ROIs

This last group focuses on identifying different histological regions or features relevant to diagnosing melanocytic tumors. Only one of the selected papers used immunohistochemical markers as the GT, in addition to the pathologist’ interpretation [22]. In this work, Alheejawi et al. proposed a two-step method to segment cutaneous melanoma regions within a WSI using MART-1 stained slide images for the GT. The authors made use of four WSIs to first identify and segment melanoma against nonmelanoma nuclei using a 25-layer CNN architecture inspired by the U-Net architecture [56], before highlighting the complete ROI by applying morphological operations to the detected nuclei. The model succeeded in segmenting the nuclei with more than 94% accuracy and segmenting the melanoma regions with a Dice coefficient of around 85%.

Liu et al. [24] used a pretrained Mask-R-CNN model to segment potential ROIs to help in melanoma diagnosis, starting by roughly identifying relevant entities before refining these and generating the final segmentation masks. The metrics were based on the pixel populations and reached an accuracy of 92%.

Additionally, Zhang et al. [26] proposed a melanoma recognition model based on the multiscale features and probability maps. The model used convolutional layers, including deformable convolution and channel attention. The proposed method could achieve 97% precision in comparison with pathologists’ labels.

In parallel, De Logu et al. [39] trained a per-patch Inception-ResNet-v2 CNN model able to discriminate, within a WSI, healthy tissues from pathological tissues in melanoma WSIs. It can recognize portions of pathological and healthy tissues on independent testing data sets with an accuracy, sensitivity, specificity, and F1-score of 96%, 95%, 97%, and 97%, respectively.

Furthermore, Kucharski et al. [23] were the first to implement a framework aiming at segmenting nests of melanocytes. In this work, the authors used a semisupervised convolutional autoencoder and leveraged both unsupervised and supervised learning to first train the encoder and decoder to reconstruct the input images and then generate masks, respectively. Despite the limited number of GT annotations, their approach reached a Dice score of 81% on the nests’ segmentation task.

Nofallah et al. [25] published the first study regarding mitotic figures in WSIs of melanoma tissue using two different state-of-the-art encoding units, ESPNet [57] and DenseNet [58], efficient spatial pyramid of dilated convolutions, and densely connected CNNs, respectively. The authors used images of mitosis and nonmitosis samples with their corresponding labels as training input. The results show a sensitivity of 97% and 96%, and specificity of 98% and 99%, respectively, with F-scores of 96% and 97%, respectively.

## 4. Discussion

To our knowledge, this SR obtained the largest number of articles (*n* = 28) published about DL models for WSIs of melanocytic tumors. Indeed, most studies analyzing skin tumors mostly focused on other image modalities such as dermoscopic or clinical images [59,60], since few research studies involving DL have yet to be carried out for skin melanocytic tumors in WSIs. These image modalities strongly differ, both in terms of the diagnostic information they provide and the technical handling they suppose (i.e., type of features, size of the images, preprocessing required, etc.). This is why this SR aims to bridge the gap in that regard, by highlighting the promise brought by DL for WSI analysis and the need for more efforts in that sense.

In this section, we describe four research tendencies where DL can be applied in dermatopathology for future ancillary tools in clinical practice. We organized them in pathologists’ comparisons with DL algorithms, diagnostic differences in a binary classification between benign and malignant tumors, prognostic approaches, and relevant histological features. Our study differentiates from other SRs, such as Haggenmuller et al. [61], focused on comparative studies between experts and the algorithm performances gathering dermoscopic, clinical, and H&E-stained WSIs. In the histopathological images group, two studies met the inclusion criteria. Zhang et al. [62] also gathered several studies, including dermoscopic images. They included five studies using AI in predicting melanoma in histopathology diagnosis and prognosis, including DL and non-DL models’ publications between 1994 and 2020. Popescu et al. [63] focused on the different neural-network-based systems explaining each one of them. They gathered studies of various data sources of WSI and non-WSI. Moreover, Cazzato et al. [64] reviewed the AI models in skin lesion images, including both melanocytic and nonmelanocytic ones.

### 4.1. Assistance Utility in Clinical Practice

All the studies analyzed in this SR achieved a promising performance according to their objectives. This would suggest that these algorithms can be helpful for clinical practice. Still, some issues must be contemplated to ensure their performance as a helpful tool in the pathology laboratory workflow and avoid diagnostic mistakes in real scenarios. In this section, we discuss the parameters that should be considered for applicability in the practice of the pathologists’ workflow, thus achieving a better generalizability performance for future research.

Regarding the number of institution sources for the data sets, 13 studies (41.38%) used only one data source. Studies with only one source of data generally used local data sets, while those using several sources tended to leverage open-access data sets as well. Despite yielding good results concerning the objective of their research, studies using only one source face a critical limitation in determining the reliability of studies in real scenarios if they were to be applied to the clinical practice. The differences in the tissues between diverse geographical and ethnic populations, or in the tissue processing (including gross sectioning, fixation, section thickness, manual or automated staining, and digitization, including scanners), can affect the image, thus resulting in differences among pathology laboratories [65]. For these reasons, in order to be efficiently applied to the clinical practice, models need to be trained with data sets coming from different sources: the more data variety a model learns from, the better it will generalize to accurately predict unseen data.

DL models are also limited, because the pathologist is the usual GT to train them. In some cases, the ambiguous characteristics of the lesion under analysis do not allow experts to perform an adequate and reliable diagnosis, as is the case for some specific melanocytic tumors. In particular, these clinically ambiguous tumors are called "melanocytic tumors of uncertain malignant potential" (MELTUMP) or "borderline", terms that are far from satisfactory or sufficient for adequate clinical management of patients [66]. Most of these tumors of uncertain malignant potential belong to spitzoid tumors. In our study, we found that only three studies included MELTUMP in their research (10.71%) [16,36,42]. Moreover, three studies used Spitz tumors in their data set (10.71%) [18,19,34], although no atypical melanocytic Spitz tumors were included. Therefore, in these types of tumors, there is a critical need for an early and more precise diagnosis to achieve the best possible clinical outcome in addition to the valuable pathologist interpretation. DL models will have to leverage pathologists’ experience combined with other biological data free of subjectivity to diagnose these tumors more accurately, such as, for example, genetics, epigenetics, survival, and outcome [64,67].

The clinical information helps obtain more accurate diagnosis in dermatopathology [68]. However, it seems that in DL methods, these variables can provoke a kind of batch effect that, instead of helping the adequate prediction of the algorithm, could affect it negatively and cause a critical pitfall during the experimental DL pipeline [69]. In one of the studies analyzed in this SR, Hohn et al. [38] showed that the clinical metadata integration to CNN did not improve the accuracy. In addition, Brinker et al. [13] showed that the combination of the model with the clinical features did not perform better compared with the one that only used image features. Instead, the other articles for prognostic purposes showed that the use of clinical variables was beneficial for their target [30,31,40,41]. Homeyer [65] established that overpassing the negative impact of these variables starts by making use of a considerable and miscellaneous data set.

### 4.2. The Rise of DL for WSI Analysis: Requirements and Promises

Numerous DL methods have been applied to medical image analysis, going from quality enhancement and filtering or Content-Based Image Retrieval (CBIR) to segmentation and classification.

In this SR, we identified relevant parameters that can affect DL models’ performance, such as the number of input WSIs, which varied a lot among studies. While the largest data set contained 981 WSIs [36], one study used only four WSIs to predict cutaneous melanoma regions [22]. In particular, in the latter, the authors extracted 100 H&E-stained images out of the original WSIs and divided them into training (70), validation (15), and testing (15) subsets. Most of the studies analyzed in this review chose high-detailed magnification levels to train their models, i.e., 40× (36%) and 20× (32%). On the contrary, low-detail magnifications, such as 10× magnification level, were used less (18%), while magnifications lower than 10× were almost never leveraged in DL studies. Two studies made use of a 5× magnification level [18,31], and one of them also leveraged the 1.25× level as well. When using these low magnification levels, studies demonstrated the benefits of combining them with higher ones, as these focus more on the lesion context.

Yet, independently of the magnification used, WSIs generally need to go through a patch extraction process in order to enable to train DL models without computational limitations. As a matter of fact, most of the studies in this SR divided WSIs into smaller patches, generally from 128 × 128 to 512 × 512 pixels each. To optimize model training, additional preprocessing steps are often applied after the patch extraction process. Since the patches containing a majority of background or blank parts might add noise to the data set, and thus hinder an optimal training, most studies applied filtering operations to discard them before feeding them to the models. In particular, we found most of the works in this SR applied the Otsu method [70] to identify tissue parts within a WSI and thus remove irrelevant patches.

Additionally, having a pool of images to train, validate, and test a model is a time-consuming and arduous process. The alternative preprocessing technique used as a general baseline in DL to provide additional samples with the intention of improving performance is data augmentation.

After applying preprocessing to the input images, most studies leveraged well-known architectures such as ResNet, VGG-series or Inception networks. Pretrained models provide a background about the general features of the image such as edge or color characteristics. This can be more efficient in time and computational costs than training a model from scratch. As mentioned earlier, WSI-based tumor analysis usually requires patch extraction to apply such models, thus dividing studies into patch-level and WSI-level analysis. In particular for diagnostic purposes, studies aiming at the latter need to aggregate patch features in order to perform the overall classification. In these works, patch aggregation can be performed in different ways and, more often than not, either involves an average of their prediction scores [13,29,35] or the majority class among patches [34]. To implement such methods, all the studies that provided the information (*n* = 11) made use of either Tensorflow or Keras with Python (46% of the computed studies that provided the information), PyTorch with Python (18% of them), or MATLAB (36%).

All this preprocessing, training, validating, and testing on histopathological patches and WSIs is not computationally compatible with CPUs. To be able to handle these heavy images in the different DL stages, significant GPU resources are required. In some articles studied in this SR, the authors reported the type of GPU used because its power can affect the DL time. The more powerful GPU can deliver the results faster and allow to consider more patches given an input WSI. GPUs are capable of handling several computations at once. Yet, even with powerful and state-of-the-art GPUs, training DL models for WSI-level analysis in recent studies in this SR still requires a previous patch extraction to avoid computational limitations. Additional improvements in GPU hardware development and distributed training procedures might help optimize DL training for WSI analysis.

### 4.3. Making the Bridge between Pathologists and AI Developers

Explainable AI (xAI) is a set of methods and processes to describe an AI model that is gaining traction in the medical field. In our SR, 42.9% of studies open the door to the explainability of the DL models by providing heat maps. Additionally, only one study [19] made use of the attention mechanism [50] in the patch aggregation step to identify patches that had more relevance in the model’s classification decision and make the final WSI-level classification. Explainable methods are increasingly in demand in the field to explain DL models’ decisions, a requirement to make them usable for further clinical use. Indeed, in clinical practice, it is transcendent for pathologists to know how these algorithms make their predictions; understanding why a system made a particular diagnosis can help convince a pathologist that it is legitimate and will help refine the prediction of the algorithm. This will promote not only the reliability of the algorithm but pave the way for other applications using AI, such as clinical trials, feedback, and teaching purposes, among others [71,72].

### 4.4. Limitations

In our study, the variety of statistical methods, the absence of available raw data, and the different DL pipelines and aims in each article limited the possibility to create a statistical comparison in a meta-analysis method.

## 5. Conclusions

DL methods have a promising future in pathologist workflow to help clinical interpretations become more objective and will likely help pathologists make more precise and reliable diagnoses. Yet, a necessary clue to make way for pathologists to integrate DL algorithms into the clinical practice is to use xAI in the studies. Indeed, in addition to allowing pathologists to trust models’ predictions, xAI will enhance the positive feedback for the algorithm to maximize its accuracy and for the pathologists to consider regions that seemed irrelevant at first according to their way of thinking.

In parallel, while most models mainly focus on the morphological features of input images, clinical information is also crucial for diagnosis and treatment. The use of such information has until now not proved to be much helpful in diagnostic studies, but it did show to be promising for prognostic purposes, using variables such as survival or the presence of tumor progression. There is a clear need for more research leveraging other sources of data such as clinical information or molecular studies, among others, and to investigate different ways to integrate them into the DL pipeline.

This exciting chapter of pathology comes with a significant challenge for expert pathologists and engineers in image analysis: the constant interaction between these two worlds. AI has the capacity to gather all types of information from different sources or studies. Its performance will improve with broader data sets, bringing unprecedented changes to the pathology field. Pathology is the bridge between clinical science and cell biology, and AI could help us to build it up.

## Abbreviations

The following abbreviations are used in this manuscript:

AI	Artificial Intelligence
AUC	Area Under the ROC Curve
CBIR	Content-Based Image Retrieval
CI	Confidence Interval
CLAIM	Checklist for Artificial Intelligence in Medical Imaging
CNN	Convolutional Neural Network
DL	Deep Learning
DNN	Deep Neural Network
DP	Digital Pathology
DSS	Disease-Specific Survival
FCN	Fully Convolutional Network
GDC	Genomic Data Commons
GT	Ground Truth
H&E	Hematoxylin and Eosin
HR	Hazard Ratio
MELTUMP	Melanocytic Tumors of Uncertain Malignant Potential
ML	Machine Learning
NCI	National Cancer Institute
PHH3	Phosphohistone-H3
RNN	Recurrent Neural Network
ROI	Region of Interest
SN	Sentinel Node
SR	Systematic Review
TCGA	The Cancer Genome Atlas
WSI	Whole-Slide Image
xAI	Explainable Artificial Intelligence
